# An Extended Thickness-Dependent Moisture Absorption Model for Unidirectional Carbon/Epoxy Composites

**DOI:** 10.3390/polym13030440

**Published:** 2021-01-30

**Authors:** Azisyahirah Azizan, Mahzan Johar, Salvinder Singh Karam Singh, Shahrum Abdullah, Seyed Saeid Rahimian Koloor, Michal Petrů, King Jye Wong, Mohd Nasir Tamin

**Affiliations:** 1 School of Mechanical Engineering, Faculty of Engineering, Universiti Teknologi Malaysia, Johor Bahru 81310, Malaysia; azisyahirah@gmail.com (A.A.); nasirtamin@utm.my (M.N.T.); 2 Faculty of Engineering and Science, Curtin University Malaysia, Miri 98009, Malaysia; mahzan.johar@curtin.edu.my; 3 Department of Mechanical and Manufacturing Engineering, Faculty of Engineering and Built Environment, Universiti Kebangsaan Malaysia, Bangi 43600, Malaysia; salvinder@ukm.edu.my (S.S.K.S.); shahrum@ukm.edu.my (S.A.); 4 Institute for Nanomaterials, Advanced Technologies and Innovation (CXI), Technical University of Liberec (TUL), Studentska 2, 461 17 Liberec, Czech Republic; s.s.r.koloor@gmail.com (S.S.R.K.); michal.petru@tul.cz (M.P.); 5 Department of Aerospace Engineering, Faculty of Engineering, Universiti Putra Malaysia, Serdang 43400, Malaysia

**Keywords:** carbon/epoxy composite, moisture absorption, non-Fickian, thickness-dependent, Weibull

## Abstract

Moisture absorption tests for materials that exhibit non-Fickian behavior generally require a relatively long period to reach saturation. Therefore, it would be beneficial to establish a relationship between the moisture content and the thickness to minimize the experimental time and cost. This research characterizes the moisture absorption behavior of AS4/8552 carbon/epoxy composites. Specimens were prepared at 4, 8, and 16 plies and immersed in distilled water at 60 °C. The relationship between the non-Fickian parameters (Fickian to non-Fickian maximum moisture content ratio *ϕ*, non-Fickian diffusivity per square thickness *α*, and non-Fickian initiation time *t_o_*) and thickness was characterized using a thickness-dependent model. A comparison with other materials revealed that all three non-Fickian parameters are able to be fitted using a power law. Nevertheless, the upper boundary for the applicability of this model was not determined in this study. The Weibull distribution plots indicate that the probability of non-Fickian moisture absorption is influenced by *ϕ* and *α* at approximately 62% within a normalized thickness range of 2–3. In regards to *t_o_*, it is 82% at a normalized thickness of 6. Therefore, the Weibull distribution is proposed for the assessment of non-Fickian moisture absorption based on the material’s thickness.

## 1. Introduction

Carbon/epoxy composites are gaining importance as aircraft materials [1,2]. When using carbon/epoxy laminated composites for aircraft applications, the materials are inevitably subjected to various environmental conditions. Upon prolonged moisture and heat attacks, the properties may degrade and lead to premature failure of the structures [3,4,5,6,7]. It is common to relate the residual properties to the absorbed moisture content of the materials [3,4,5]. Therefore, before characterizing the mechanical properties, studying the materials’ moisture absorption behavior is essential. The relationship between the residual properties and moisture content can be established through the moisture uptake characteristics. Therefore, the mechanical properties at any instant can be predicted.

Accelerated tests are usually conducted in laboratories to quantify the moisture uptake in materials. However, even with accelerated tests, a long period of time may still be required to reach saturation. For example, a 16-ply carbon/epoxy composite with a thickness of 3.2 mm, immersed in distilled water at 70 °C, took more than nine months to reach saturation [8]. Additionally, at an aging condition of 50 °C/95% relative humidity (RH), Davidson et al. [9] immersed thermoplastic particles in toughened carbon/epoxy composites for around 320 days before conducting the delamination tests. Moreover, LeBlanc and LaPlante [10] reported that approximately 11 months were required for carbon/epoxy composites that were aged in distilled water at an aging temperature of 70 °C to reach saturation. Zhao et al. [11] reported that for 8-harness satin-weave glass/bismaleimide composites submerged in seawater at 50 and 80 °C, saturation was achieved after an ageing period of more than 8 months. An alternative for reducing the amount of time required for saturation is to conduct the moisture absorption test using thinner specimens. Nevertheless, many polymer-based materials exhibit non-Fickian behavior. For non-Fickian behavior, the moisture absorption varies with thickness. Therefore, the moisture absorption curves obtained at a smaller thickness cannot be used for thicker composites in real-life applications.

Among the non-Fickian models that are available in the literature, one of them is Bao’s model. This model was developed to describe the long-term water uptake behavior in bismaleimide (BMI) resin immersed in distilled water at 70 °C [12]. This model was found to fit linear non-Fickian behavior well and has been employed to describe the non-Fickian moisture absorption behavior of 3-ply and 12-ply uni-weave carbon/BMI composites [13], 3-ply woven and hybrid carbon/BMI composites [14], pultruded unidirectional carbon/epoxy composites [15], E-glass (E-8204)/epoxy (DER383) reinforced with Cloisite 30B and Cloisite 10A montmorillonite composites [16], and pure and carbon nanotube reinforced flax/epoxy composites [17]. Another popular model is the two-stage diffusion model proposed by Berens and Hopfenberg [18], which considers multiple viscoelastic processes within the polymer structure. This model has been used to describe the non-Fickian moisture absorption in E-glass/BMI composites [11,19]. Moreover, the Langmuir model which was proposed by Cater et al. [20] considers the probability of free and bound water molecules. This model has been adopted by various researchers when characterizing the non-Fickian moisture absorption behavior of FM300 epoxy adhesive [21], DGEBA-based epoxy adhesives [22], Fiberite ANC3K/948A1 graphite/epoxy composites [23], EC 2216 epoxy adhesive [24], Hysol EA9360 adhesive and T-300 plain weave reinforced epoxy composites [25], and biphenyl epoxy molding compounds [26]. The parallel Fickian model is another common model employed for non-Fickian behavior, where the moisture absorption is described by the summation of two Fickian models. This model has been employed in a rubber toughened epoxy adhesive (Araldite 2007) [27] and epoxy molding compounds (EMCs) [26,28,29]. Nevertheless, the abovementioned non-Fickian models are limited to the specific experimental conditions designed by the authors. Considering this, there is a need to investigate the relationship between the non-Fickian moisture absorption characteristics and the thickness of materials. If this relationship can be established, the experimental moisture absorption tests can be minimized. A thickness-dependent non-Fickian moisture absorption model has been proposed and further generalized [30,31]. However, the range of the applicability of the thickness-dependent model has not yet been assessed. Therefore, it is necessary to perform moisture absorption tests at a greater thickness to evaluate the upper boundary of the model.

This study aims to assess the applicability of the thickness-dependent non-Fickian moisture absorption model at a greater thickness. Continuous water absorption tests in distilled water at a constant temperature of 60 °C were conducted for AS4/8552 carbon/epoxy composites at 4, 8, and 16 plies. Subsequently, the fictitious Fickian curve was plotted. Then, the non-Fickian parameters were evaluated, and the applicability of the thickness-dependent model with a 16-ply composite was investigated. Weibull distribution plots were also modeled to evaluate the non-Fickian parameters’ sensitivity to non-Fickian behavior, in order to predict an appropriate thickness for the investigated materials.

## 2. Materials and Methods

### 2.1. Materials and Specimens

The material used in this study was an AS4/8552 carbon/epoxy composite. According to the manufacturer, this composite is employed in primary aerospace structures due to its good impact resistance and damage tolerance. The fiber and resin density is 1.79 and 1.30 g/cm^3^, respectively. The nominal fiber volume fraction is 57.42%, and the nominal ply thickness is 0.15 mm. Three laminates were fabricated, with 4, 8, and 16 plies, with a nominal thickness of 0.6, 1.2, and 2.4 mm, respectively. All laminates were fabricated at Composites Technology Research Malaysia (CTRM) using an autoclave with the recommended manufacturing cycle.

### 2.2. Moisture Absorption Test

**Figure 1:**
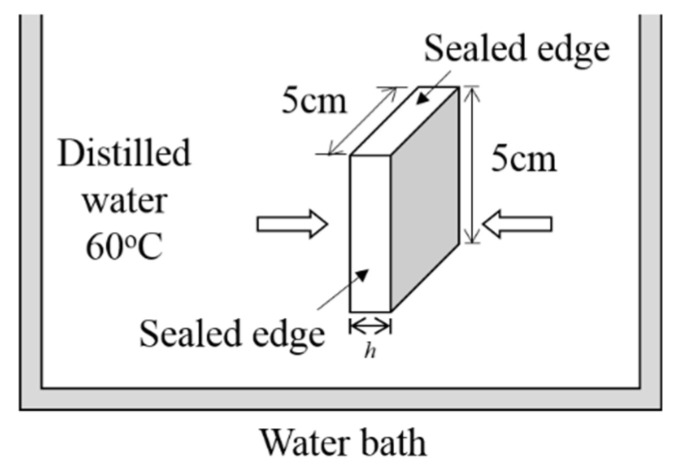
Figure illustrating the specimens’ configuration and moisture absorption condition.

Each laminate was cut into six specimens with a 5 × 5 cm^2^ size. The edges of all specimens were coated with water-resistant paint to avoid water penetration from the sides. Subsequently, the initial weight of all specimens was measured using a Shimadzu ATY224 four-decimal digital balance. They were subjected to continuous distilled water immersion in an HH-6 thermostatic water bath at a fixed temperature of 60 °C. Weight gains of all specimens were measured periodically. For each reading, three measurements were made to obtain the average moisture content, *M*. Figure 1 illustrates the specimens’ configuration and the aging condition for the moisture absorption test.

## 3. Thickness-Dependent Moisture Absorption Model

**Figure 2:**
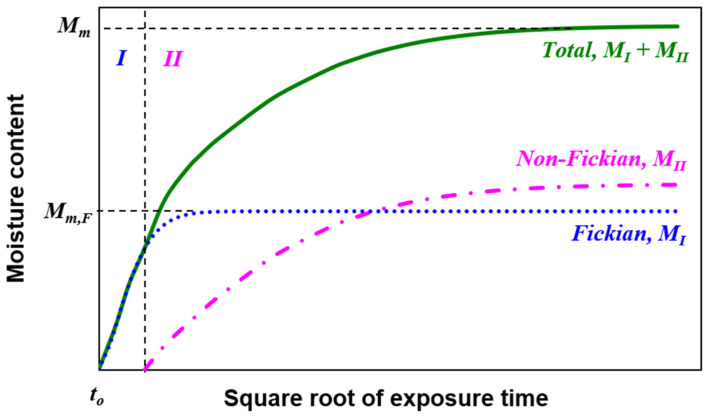
Moisture absorption distribution of the thickness-dependent moisture absorption model.

Figure 2 illustrates the two stages of the thickness-dependent non-Fickian moisture diffusion model used in this study. The model assumes that, during Stage I, water molecules are free to move into the free space in the epoxy. Therefore, the water uptake behavior follows Fickian diffusion behavior. During Stage II, non-Fickian dominates, where the water absorption rate worsens due to swelling and the comparable relaxation rate of the polymer [30].

For one-dimensional moisture absorption with a fixed aging temperature and the absence of the initial moisture content, this thickness-dependent non-Fickian moisture absorption model is as follows [30,31]:

(1) $$M(t)=M_{I}(t)+M_{II}(t)=\phi M_{m}\left\{1-\exp\left[-7.3{\left(\frac{D_{z}t}{h^{2}}\right)}^{0.75}\right]\right\}+(1-\phi )M_{m}\left[1-\left\{\exp-\left[{\left(\alpha \langle t-t_{o}\rangle \right)}^{0.75}\right]\right\}\right]$$

In Equation (1), *M_m_* means the maximum moisture content of each specimen, *D_z_* refers to the Fickian diffusivity of the material, *h* indicates the thickness of the specimens, and *t* is any instant of the immersion time. Additionally, *ϕ* represents the ratio of the maximum moisture content at Stage I to the total maximum moisture content of the specimen (*ϕ = M_m,F_/M_m_*), *α* is the non-Fickian diffusivity per square thickness, and *t_o_* indicates the initiation time of Stage II. The Macaulay bracket, < > for the time delay term, <*t* − *t_o_*>, depicts that non-Fickian behavior only occurs after *t* ≥ *t_o_*. For this model to be applicable, Stage I of the experimentally reduced moisture absorption curves (*M* vs. *√t/h*) at all thicknesses must overlap. This behavior is observed because Stage I is assumed to exhibit Fickian diffusion behavior.

## 4. Experimental Results and Discussion

### 4.1. Moisture Absorption Curves

**Figure 3:**
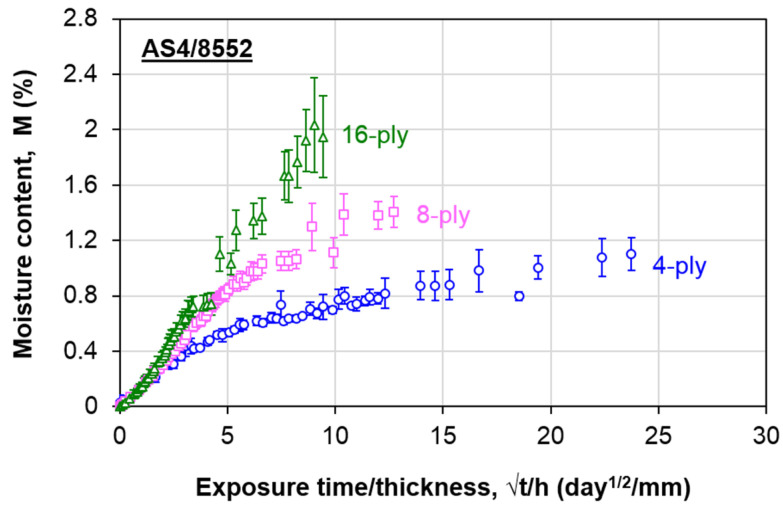
Water absorption behavior of AS4/8552 carbon/epoxy composite laminates.

Figure 3 compares the moisture absorption behavior of the composite at all thicknesses. Each data point refers to the average value of six specimens, and the error bars indicate the standard deviation of each data point. More data are considered at the beginning due to the more rapid moisture absorption rate. For 4-ply and 8-ply composites, the moisture content was measured for approximately six months. As for the 16-ply composite, the measurement was carried out for around 11 months. The moisture uptake exhibits similar behavior at the initial stage. This behavior implies Fickian diffusion. At the later stage, the water ingression deviates among laminates with different plies, indicating the non-Fickian behavior. The overall moisture absorption behavior follows the same trend described by the thickness-dependent moisture absorption model, where the moisture content increases with the thickness. This phenomenon is postulated to be due to the interaction of the bound molecules with the reaction sites of the epoxy chain, which enhances the moisture absorption [32]. Therefore, the experimental data were further analyzed to determine the non-Fickian AS4/8552 carbon/epoxy composite parameters.

### 4.2. Fictitious Fickian Diffusion Curve

**Figure 4:**
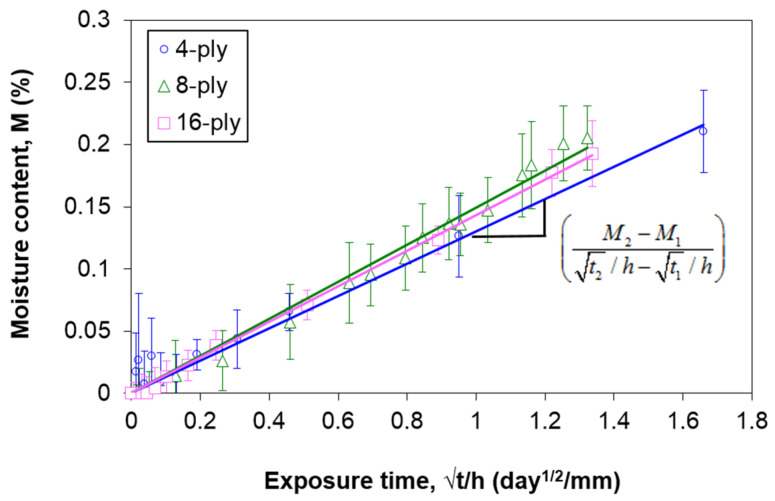
Determination of the slope of the initial linear region for specimens at all thicknesses.

To apply the aforementioned thickness-dependent moisture absorption model, a specific thickness that follows Fickian diffusion is required. Previously, it has been demonstrated that it is suitable to regard a single-ply composite as the thickness that exhibits Fickian behavior, even without experimenting [31]. The single-ply composite only requires a short aging duration to achieve saturation. The high scatter in the weight gain results among different specimens is generally attributed to the low initial weight. Additionally, if the saturation is achieved in a brief period, there is insufficient time to obtain enough data to plot the moisture absorption curve. These could be the reasons why ASTM D5229 [33] recommends a minimum weight of 5 g. In this regard, the moisture absorption behavior in the single-ply carbon/epoxy composite used in this study was modeled using the fictitious curve concept described in previous works [30,31]. The steps are as follows:

- From the reduced moisture absorption curves (*M* versus *√t/h*) (Figure 3), identify the initial overlapping region among all thicknesses (Figure 4), which is regarded as the Stage I region defined in Figure 2;
- The deviation of the reduced experimental curves in this study was estimated to be 60% of *M_m,F_*. From there, estimate the average *M_m,F_* for the material (Table 1). A coefficient of variation (C.V) of 8.11% signifies good repeatability of the results;
- Calculate the average slope of the linear region from all experimental curves (Figure 4 and Table 1). For all three sets of data, *R*^2^ is at least 0.98, and the C.V. of the slopes is within 10%. These data suggest that the estimated average slope value is reliable;
- Determine the apparent diffusivity, *D_z_*, of the fictitious Fickian curve using Equation (2). For the composite used in this study, *D_z_* is estimated to be 4.25 × 10^−2^ mm^2^/day;
  (2) $$D_{z}=\pi {\left(\frac{1}{4M_{m,F}}\right)}^{2}{\left(\frac{M_{2}-M_{1}}{\sqrt{t_{2}}/h-\sqrt{t_{1}}/h}\right)}^{2}$$
- Plot the fictitious Fickian curve (which is the first term in Equation (1)) using Equation (3). For the AS4/8552 carbon/epoxy composite, the unit ply thickness *h_F_* = 0.15 mm.
  (3) $$M_{I}(t)=M_{m,F}\left\{1-\exp\left[-7.3{\left(\frac{D_{z}t}{h_{F}^{2}}\right)}^{0.75}\right]\right\}$$

### 4.3. Determination of Non-Fickian Parameters

**Figure 5:**
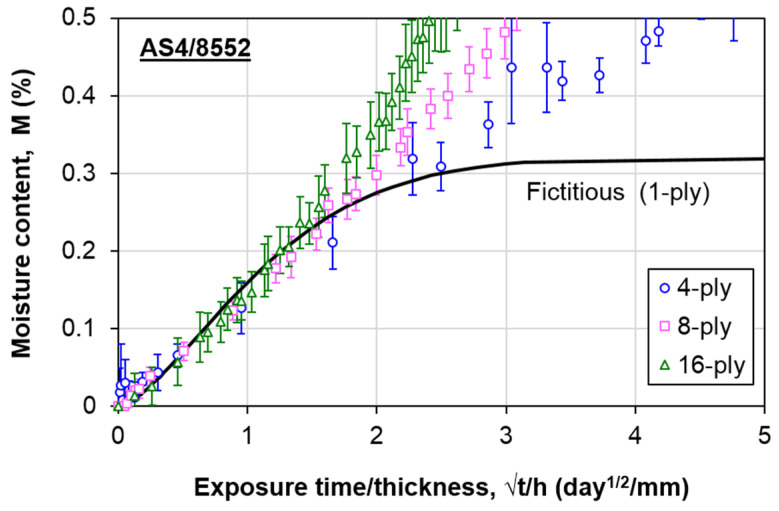
Estimation of the initiation of non-Fickian behavior.

**Figure 6:**
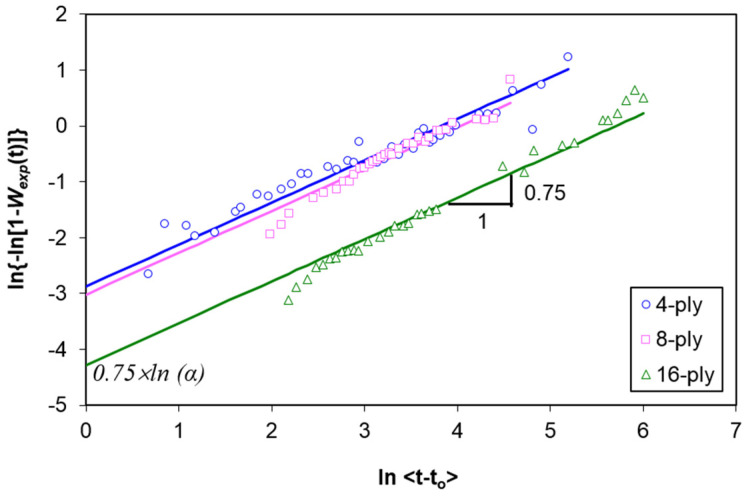
Illustration of how non-Fickian parameters are determined.

Subsequently, the non-Fickian parameters (*ϕ*, *α,* and *t_o_*) are determined as follows:

- Calculate *ϕ = M_m,F_/M_m_* for each thickness;
- Plot the fictitious Fickian curve, together with the experimental curves (Figure 5). Estimate the deviation point (*√t_o_/h*) from the figure. From there, determine *t_o_* for each thickness;
- Subtract the experimental moisture content *M*(*t*) from the analytical *M_I_*(*t*) (which is determined in the previous section). This step gives the experimental *M_II_*(*t*) term;
- Plot the experimental *W*(*t*) (denoted as *W_exp_*(*t*)) using Equation (4) below:
  (4) $$W_{\exp}(t)=\frac{M(t)-M_{I}(t)}{M_{m}-M_{m,F}}$$
- Apply logarithms of both sides twice for the analytical *W*(*t*) term:
  (5) $$\ln\{-\ln[1-W(t)]\}=0.75\times \ln\langle t-t_{o}\rangle +0.75\times \ln\alpha$$
- Plot the curve of ln[−ln[1 − *W_exp_*(t)]] versus 0.75 × ln<*t* − *t_o_*>, and fit the data with a straight line. From the ordinate intersection (0.75 × ln *α*), determine the value of *α*. As an example, Figure 6 labels the slope and *y*-intercept of the 16-ply composite.

**Figure 7:**
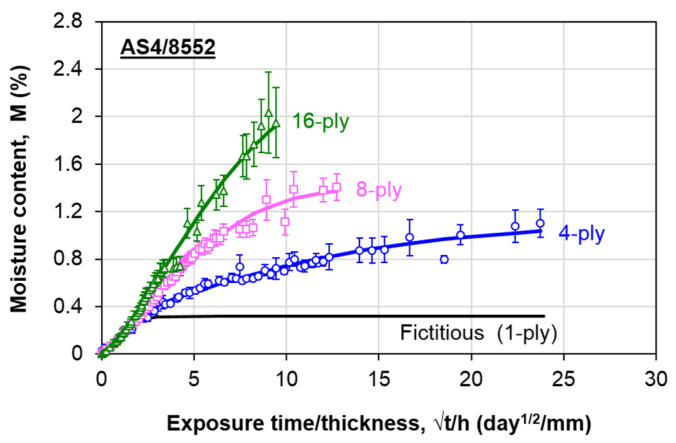
Experimental and fitted water absorption behavior of AS4/8552 carbon/epoxy composite laminates.

Following this approach, all necessary parameters, as listed in Equation (1), are obtained. Table 2 lists the non-Fickian parameters at all thicknesses. The fitted-curves are plotted in Figure 7 by the solid lines. The results show good fits of the experimental data using the thickness-dependent moisture absorption model.

### 4.4. Generalization of Non-Fickian Parameters

The non-Fickian parameters were further normalized to compare them with other materials that have been studied previously [30,31]. The thickness was also normalized with respect to the Fickian thickness, *h_F_*. For *ϕ*, it is already in the normalized form (*ϕ = M_m,F_/M_m_*). Both *α* and *t_o_* were normalized with respect to the normalized thickness values, where *h’* = 2. The value of *h’* = 1 corresponds to the Fickian behavior, so *α* and *t_o_* (which are non-Fickian parameters) do not exist.

**Figure 8:**
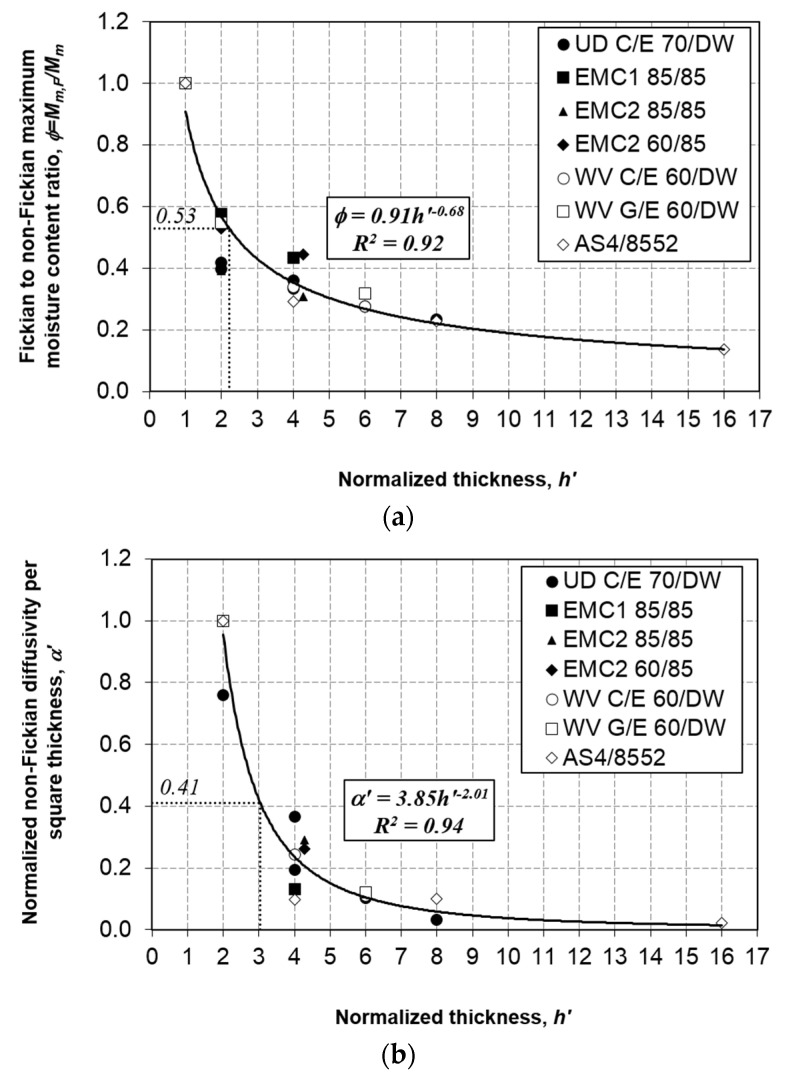

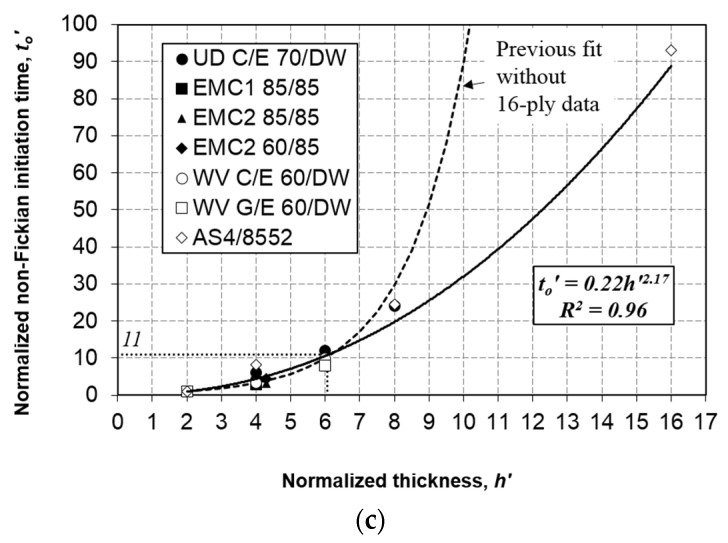
Variation of the non-Fickian parameters with the normalized thickness: (**a**) Fickian to non-Fickian maximum moisture content ratio *ϕ*; (**b**) normalized non-Fickian diffusivity per square thickness *α’*; and (**c**) normalized non-Fickian initiation time *t_o_’*.

From Figure 8a,b, it was found that both *ϕ* and *α’* follow a trend similar to that of other materials that have been studied previously [30,31]. This parameter includes the extended normalized thickness (*h’* = 16) in this study. Nevertheless, for *t_o_’* (Figure 8c), it was noticed that the previous fitted relationship as described in reference [31] does not fit the value at *h’* = 16. Therefore, the data were refitted using the power law to give a better fit. The best-fit parameters for all three non-Fickian parameters are shown in Figure 8. The regression fit, *R*^2^, of at least 0.92 suggests a good fit for all three parameters’ plots. Furthermore, from Figure 8c, it is apparent that the upper limit of *t_o_* has not yet been determined. More works are needed to determine the upper boundary of this parameter.

### 4.5. Characterization of the Distribution Model for Non-Fickian Parameters

**Figure 9:**
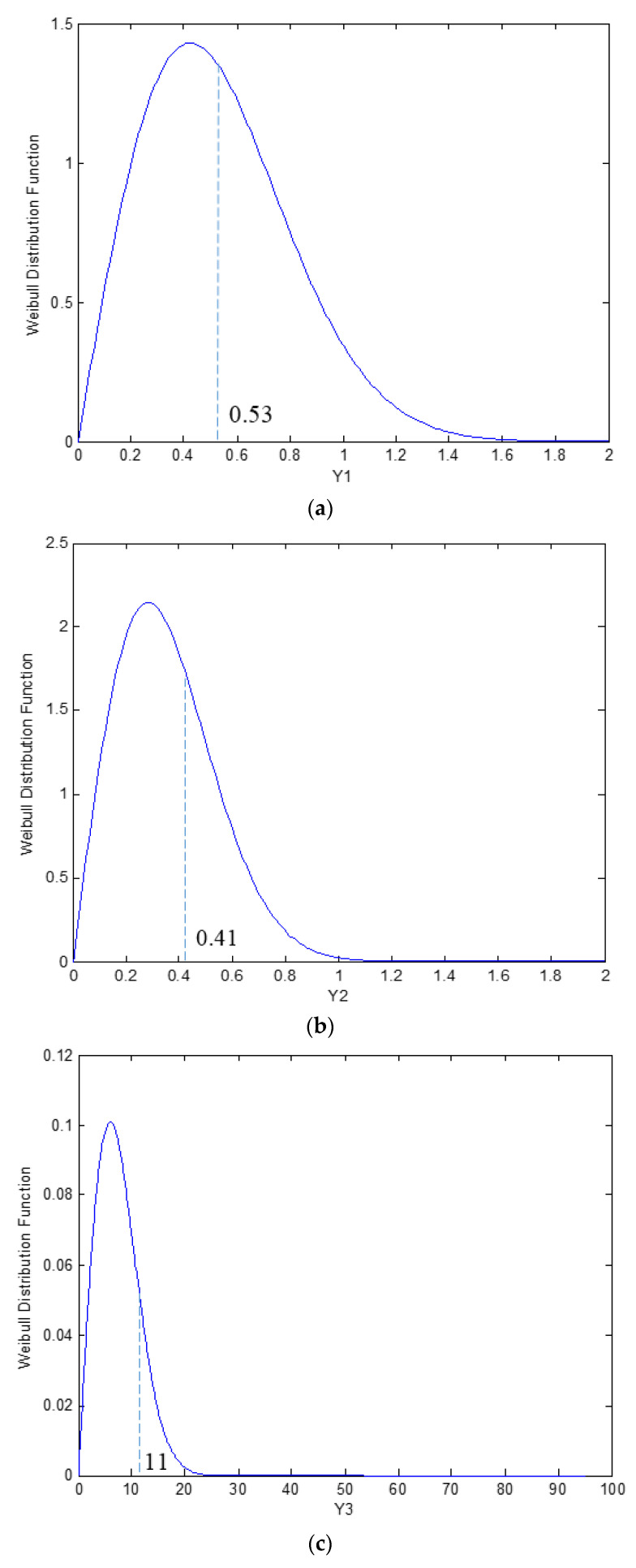
The probability distribution function of non-Fickian parameters for the normalized thickness: (**a**) Fickian to non-Fickian maximum moisture content ratio *ϕ*; (**b**) normalized non-Fickian diffusivity per square thickness *α’*; and (**c**) normalized non-Fickian initiation time *t_o_’*.

The Weibull distribution is proposed in this study to assess the distribution of non-Fickian parameters (*ϕ*, *α*, and *t_o_*) obtained from the experimental data by extracting the statistical features using the probability distribution function (PDF) and cumulative distribution function (CDF). Therefore, the Weibull distribution quantifies and control the uncertainties of the non-Fickian parameters in moisture absorption behavior based on the materials’ normalized thickness. From the non-Fickian two-parameter Weibull probability distribution shown in Figure 9, the mean normalized values of *ϕ* = 0.53, *α’* = 0.41, and *t_o_’* = 11 were obtained (marked in Figure 8).

**Figure 10:**
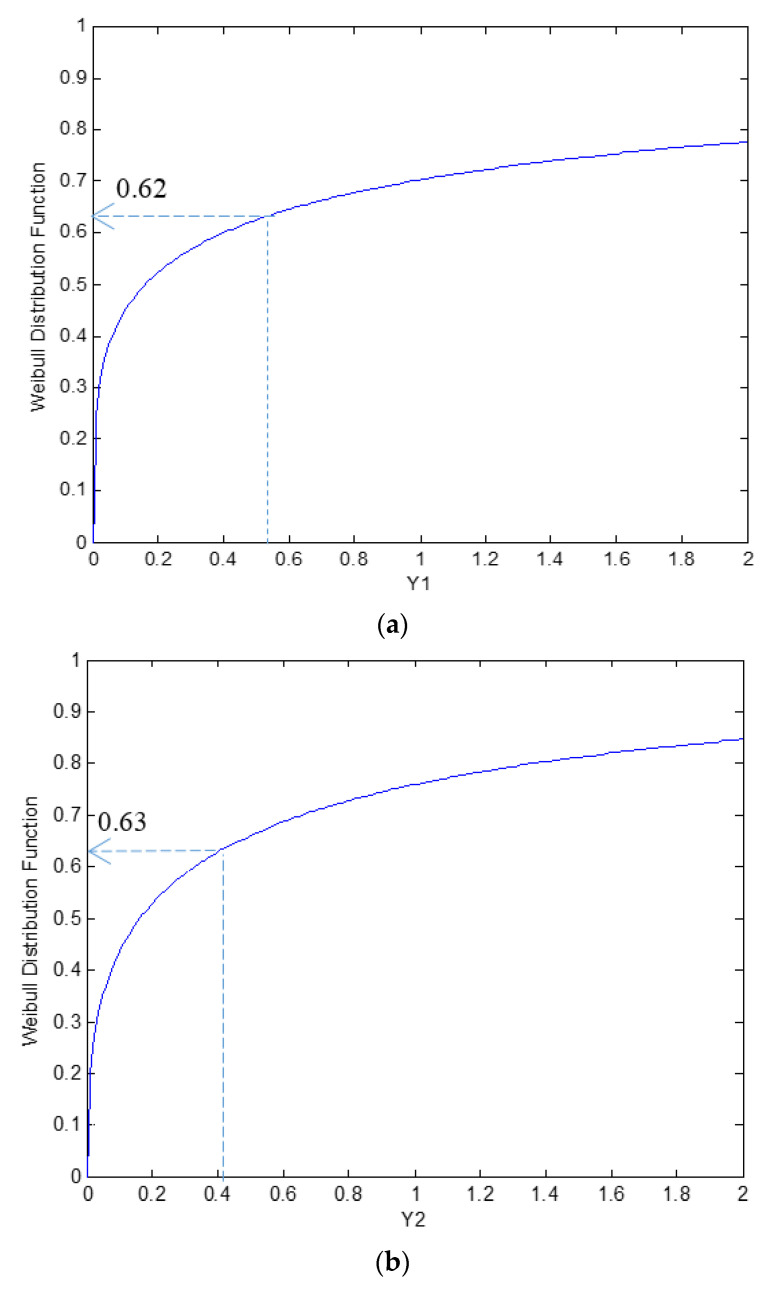

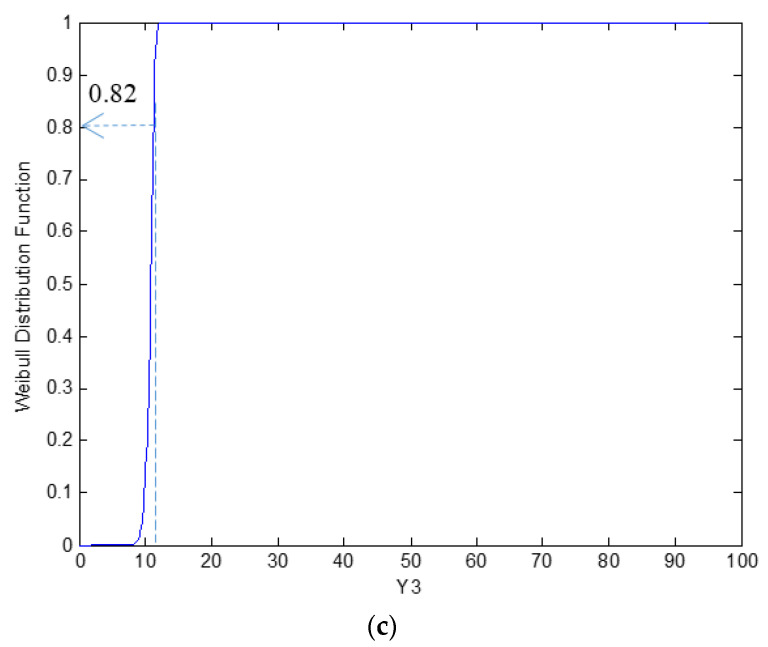
The cumulative distribution function of non-Fickian parameters for the normalized thickness: (**a**) Fickian to non-Fickian maximum moisture content ratio *ϕ*; (**b**) normalized non-Fickian diffusivity per square thickness *α’*; and (**c**) normalized non-Fickian initiation time *t_o_’*.

The mean data points were used to assess the probability of non-Fickian moisture absorption from the cumulative distribution function based on the materials’ normalized thickness, as illustrated in Figure 10. Using the average non-Fickian parameters, the Weibull CDF indicates that *ϕ* and *α’* have the lowest probability at approximately 0.62 compared with *t_o’_*, estimated at 0.82. This result suggests that the probability of non-Fickian moisture absorption is mainly influenced by *ϕ* and *α* at approximately 62% within a range of normalized thickness *h’* = 2–3. Additionally, it is affected by the non-Fickian initiation time, *t_o_*, at around 82%, with a normalized thickness *h’* = 6 (refer to Figure 8 for the corresponding normalized thickness).

## 5. Conclusions

This study characterized the water uptake behavior in unidirectional AS4/8552 carbon/epoxy composites at 4, 8, and 16 plies. All specimens were continuously submerged in distilled water at 60 °C. Through fitting the fictitious Fickian curve, it was estimated that the apparent diffusivity *D_z_* = 4.25 × 10^−2^ mm^2^/day and the single-ply maximum moisture content *M_m,F_* = 0.322%. By implementing the thickness-dependent moisture absorption model, the relationships between the non-Fickian parameters and normalized thickness were further generalized and extended to a larger normalized thickness (*h’* = 16). Specifically, *ϕ* = 0.91*h*’^−0.68^, *α’* = 3.85*h’*^−2.01^, and *t_o_’* = 0.22*h’*^2.17^. Generalization of the non-Fickian parameters is beneficial for minimizing the amount of experimental work. Furthermore, Weibull distribution plots suggest that the probability of non-Fickian moisture absorption is mainly affected by *ϕ* and *α* at approximately 62% for a normalized thickness *h’* = 2–3. Moreover, the non-Fickian initiation time *t_o_* results in approximately 82% absorption when *h’* = 6. Further works are required to determine the upper limit of the thickness-dependent model.

## Figures and Tables

**Table 1:** Fictitious Fickian parameters of AS4/8552 carbon/epoxy composites.

No. of Ply	*M_m,F_*	Slope, (*M*_2_ − *M*_1_)/(*√t*_2_/*h* − *√t*_1_/*h*)	*R* ^2^
4	0.319	0.1356	0.9843
8	0.297	0.1486	0.9957
16	0.349	0.1646	0.9801
Average	0.322	0.1496	
S.D	0.0261	0.0145	-
C.V (%)	8.11	9.71	-

**Table 2:** Non-Fickian parameters of AS4/8552 carbon/epoxy composites.

	*h* (mm)	0.6	1.2	2.4
Parameter				
*ϕ*		0.29	0.23	0.14
*α* (10^−3^ day^−1^)		19.3	20.1	4.2
*t_o_* (hours)		24	71	269

## Data Availability

Not applicable.
